# JOA: Joint Overlap Analysis of multiple genomic interval sets

**DOI:** 10.1186/s12859-019-2698-4

**Published:** 2019-03-08

**Authors:** Burçak Otlu, Tolga Can

**Affiliations:** Department of Computer Engineering, Middle East Technical University, Ankara, 06800 Turkey

**Keywords:** Genome analysis, Joint overlap analysis, Interval overlap, Interval intersection, Genomic interval intersection, Segment tree, Indexed segment tree forest, Space partitioning algorithms

## Abstract

**Background:**

Next-generation sequencing (NGS) technologies have produced large volumes of genomic data. One common operation on heterogeneous genomic data is genomic interval intersection. Most of the existing tools impose restrictions such as not allowing nested intervals or requiring intervals to be sorted when finding overlaps in two or more interval sets.

**Results:**

We proposed segment tree (ST) and indexed segment tree forest (ISTF) based solutions for intersection of multiple genomic interval sets in parallel. We developed these methods as a tool, Joint Overlap Analysis (JOA), which takes *n* interval sets and finds overlapping intervals with no constraints on the given intervals. The proposed indexed segment tree forest is a novel composite data structure, which leverages on indexing and natural binning of a segment tree. We also presented construction and search algorithms for this novel data structure. We compared JOA ST and JOA ISTF with each other, and with other interval intersection tools for verification of its correctness and for showing that it attains comparable execution times.

**Conclusions:**

We implemented JOA in Java using the fork/join framework which speeds up parallel processing by taking advantage of all available processor cores. We compared JOA ST with JOA ISTF and showed that segment tree and indexed segment tree forest methods are comparable with each other in terms of execution time and memory usage. We also carried out execution time comparison analysis for JOA and other tools and demonstrated that JOA has comparable execution time and is able to further reduce its running time by using more processors per node. JOA can be run using its GUI or as a command line tool. JOA is available with source code at https://github.com/burcakotlu/JOA/. A user manual is provided at https://joa.readthedocs.org

**Electronic supplementary material:**

The online version of this article (10.1186/s12859-019-2698-4) contains supplementary material, which is available to authorized users.

## Background

Genomic interval intersection is a major component of analysis pipelines for Next Generation Sequencing (NGS) technologies such as RNASeq, ChIPSeq, and exome sequencing. There are various existing tools that perform interval intersection [1–5] and many other genomic analyses. UCSC Genome Browser has been continuously improved since its first launch. Lately, the Data Integrator feature is released in UCSC Genome Browser, which allows users to combine and extract data from multiple tracks (up to 5 tracks), simultaneously [1]. BEDTools is a toolset developed for genomics analysis tasks such as comparison, manipulation, and annotation of genomic features in BAM, BED, GFF and VCF formats [2]. Pybedtools extends upon BEDTools and provides a Python interface for BEDTools [3].

BEDOPS is a highly scalable and easily-parallelizable genome analysis toolkit, which enables tasks to be easily split by chromosome for distributing whole-genome analyses across a computational cluster [4]. BEDOPS requires sorted interval set files before intersection. GROK utilizes region algebra for genomic region operations and provides R, Python, LUA, command line interfaces, and C++ API extension [5]. NCList uses a novel data structure named Nested Containment List (NCList) for interval databases [6]. NCList keeps intervals in lists such that intervals in each list have containment relation. However, no list fully contains one another, which implies that if lists are ordered on their start positions, then they are also sorted on their end positions. GLANET stands for genomic loci annotation and enrichment tool and provides joint overlap analysis of 2 to 3 tracks at most [7].

Layer et al. propose a novel parallel “slice-then-sweep” algorithm for *n*-way interval set intersection. Their algorithm requires the input intervals not to be contained fully in one another [8]. Tabix indexes tab-delimited files and converts a sequential access file into a random access file [9].

In this paper, we generalize the problem of genomic interval intersection (finding common overlapping intervals) from 2 or 3 interval sets to *n* interval sets. We implement segment tree based construction and search algorithms in parallel for each interval set and chromosome. We accept *n* interval sets in Browser Extensible Data (BED) format and we apply a divide and conquer algorithm for finding common overlapping intervals. The intervals in a single set may overlap, may contain one another, and may be unsorted. We divide the interval sets into two sets, recursively, until one or two interval sets remain as base cases. In case of two interval sets, we construct a segment tree from one of the interval sets and we then search the intervals of the other interval set as query intervals on the constructed segment tree. In case of one interval set, we return all of its intervals as overlapping intervals. Results coming from the base cases are combined as follows: one of the results is used as query intervals, a segment tree is built on the other result, query intervals are searched on the segment tree, and overlapping intervals are returned. This process is repeated recursively until all of the *n* interval sets are processed.

**Fig. 1:**
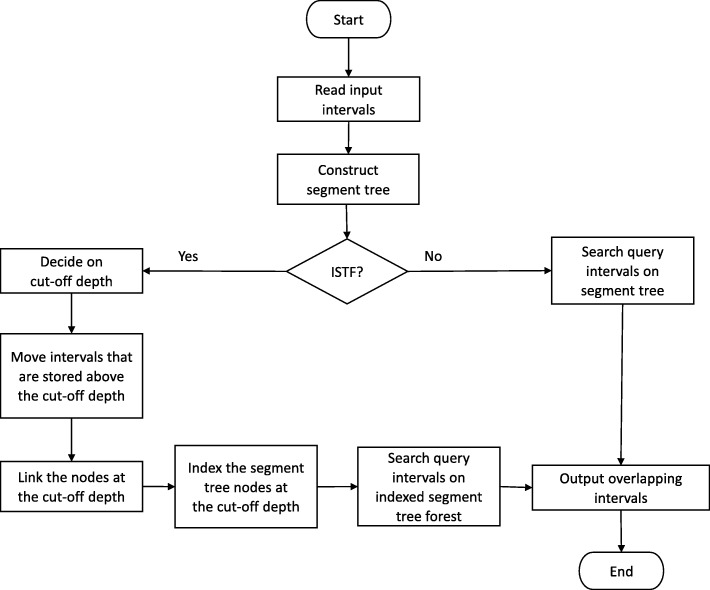
Work-flow for finding jointly overlapping intervals of *n* interval sets, displays the proposed data structures: segment tree and indexed segment tree forest, and the key steps on them

We also propose a composite data structure, indexed segment tree forest which leverages on natural binning of segment trees and indexing the segment tree nodes at a certain depth. The same divide and conquer algorithm is also applied on the indexed segment tree forest (ISTF). However, this time when a segment tree is constructed, we proceed by converting this segment tree to an indexed segment tree forest by cutting this tree at a certain depth and indexing the segment tree nodes at that cut-off level. Our aim is to reduce the search space and search on the shorter indexed segment tree forest rather than one tall segment tree and eventually to reduce the query time. The overview of the proposed methodology is depicted in Fig. 1.

## Results

### Execution time comparison

We compared JOA with other tools using their latest available versions such as GROK v1.1.1, BEDTools v2.27.1, and BEDOPS 2.4.35. To compare the tools, we have used real and semi-synthetic datasets. As an additional case study, we compared JOA segment tree and JOA indexed segment tree forest using 141 ENCODE Dnase hypersensitive sites. Details of these datasets can be found in Availability of data and material.

During comparisons, JOA is run both with segment tree (ST) and indexed segment tree forest (ISTF) options, GROK is run through its python API and intersectionL method is utilized, BEDTools is called by its intersect -a -b utility, and for BEDOPS, –intersect set operation is used. BEDTools is run with its “multiinter” parameter when there are more than 2 input files. BEDTools gives a number to each input file starting at 1, when run with the “multiinter” parameter and produces a comma separated list of the input file numbers that are jointly overlapping in the fifth column of its output file. Therefore, from BEDTools’ output file, we counted the number of rows that has all the file numbers separated by comma in the fifth column and calculated the number of jointly overlapping intervals.

All of the runs are carried out on Centos 6.6, Intel Xeon Gold 6132 (skylake), 2.6GHz ×28 processors (14 cores/socket). All the provided execution times in Tables 1, 2, 3 and 4 are averaged over 50 runs.

**Table 1:** Average execution time comparison of the tools for the six benchmarks

Benchmarks	Average execution times in seconds						
	(1n,1ppn)					(1n,8ppn)	
	GROK	BEDOPS	BEDTools	JOA ST	JOA ISTF	JOA ST	JOA ISTF
2 small files	1.1426	0.1785	0.4204	1.5135	1.7880	0.8363	0.9712
5 small files	2.3757	0.3104	12.6509	2.1504	2.7396	1.2470	1.5531
(2.3M, 2.3M)	13.5173	1.8656	14.4679	16.1611	17.0239	9.5708	9.7355
(6.4M, 6.4M)	34.2736	4.8053	63.8499	39.7799	43.5383	23.9280	24.0116
(9.2M, 9.2M)	54.5452	7.1102	65.3324	56.0001	55.9838	31.7290	32.5613
(6.4M, 9.2M)	49.2007	5.4660	43.1104	48.0046	47.9815	23.2048	24.3927

GROK, JOA, BEDTools and BEDOPS execution times are averaged over 50 runs

**Table 2:** Number of interval files are increased by two fold whereas number of intervals in each file is kept constant

Simulations 1^*st*^ Scenario (#ofFiles,#ofIntervals)	Average execution times in seconds						
	(1n,1ppn)					(1n,2ppn)	
	GROK	BEDOPS	BEDTools	JOA ST	JOA ISTF	JOA ST	JOA ISTF
(2,100000)	0.6973	0.0366	0.2380	1.0846	1.2861	0.6677	0.7121
(4,100000)	1.3527	0.0397	3.8666	1.4480	1.7342	1.0410	1.0932
(8,100000)	2.7232	0.0447	8.2620	2.0175	2.4720	1.4686	1.5197
(16,100000)	5.7325	0.0565	18.9487	2.8402	3.6387	2.0252	2.3860
(32,100000)	12.5799	0.0819	45.7564	4.4840	5.4132	3.0647	3.6630
(64,100000)	30.2584	0.1283	116.7736	6.9214	7.9966	4.5746	5.4844
(128,100000)	66.7122	0.2131	312.8438	12.1682	13.6740	7.4285	8.3240
(256,100000)	-	0.4229	891.2454	22.6010	24.0628	15.3915	16.2595
(512,100000)	-	0.8283	2887.3816	42.0187	45.3321	26.9868	28.8161

Average execution time comparison of the tools for the first scenario using semi-synthetic datasets. GROK, JOA, BEDTools and BEDOPS execution times are averaged over 50 runs except BEDTools runs for 128, 256 and 512 files which are averaged over 2 runs

**Table 3:** Number of intervals in each file is increased by two fold whereas number of interval files is kept constant

Simulations 2^*nd*^ Scenario (#ofFiles,#ofIntervals)	Average execution times in seconds						
	(1n,1ppn)					(1n,2ppn)	
	GROK	BEDOPS	BEDTools	JOA ST	JOA ISTF	JOA ST	JOA ISTF
(2,1M)	9.0330	0.3635	4.2109	4.5382	5.2108	3.4880	4.1231
(2,2M)	19.7827	0.6202	11.4695	11.8475	12.2080	7.2478	8.2213
(2,4M)	44.9517	1.8149	33.1372	29.9437	32.5128	18.4192	19.3616
(2,8M)	102.6299	2.9645	108.0360	73.2382	74.9382	55.1262	56.9878
(2,16M)	255.6354	5.7609	397.5899	190.2903	192.3511	178.7225	185.8660

Average execution time comparison of the tools for the second scenario of semi-synthetic datasets. GROK, JOA, BEDTools and BEDOPS execution times are averaged over 50 runs

**Table 4:** Average execution time comparison of JOA ST and ISTF for 141 interval sets of Dnase hypersensitive sites

ENCODE 141 DHSs files	Average execution times in seconds	
	(1n,1ppn)	(1n,8ppn)
JOA ST	15.8894	9.6301
JOA ISTF	19.0009	10.6943

JOA ST and ISTF execution times are averaged over 50 runs. Average execution times are reduced as number of processors per node is increased from 1 to 8

There exists other tools and formats such as BGT, GQT, SeqArray and BGEN [10–13] that requires joint overlap analysis. BGT is a new, compact file format for efficiently storing and querying whole-genome genotypes of tens to hundreds of thousands of samples. BGT search alleles/variants in the input bgt format file within a given site or list of sites [10]. Genotype Query Tools (GQT) is a new indexing strategy that expedites analyses of genome variation datasets in VCF format based on sample genotypes, phenotypes and relationships [11]. SeqArray is a storage-efficient high-performance new data format for WGS variant calls which comes with SeqArray software package including key R functions [12]. BGEN is another novel binary file format for imputed genotype and haplotype data which are supported by many tools[13]. BGT, SeqArray and BGEN introduce their own new data format and carry out analysis on their new data formats except GQT, which carries out analysis on the VCF format. However, JOA does not support these data formats but designed for text based formats including “.bed” and “.pk’ formats. Therefore, we did not compare JOA against them.

#### Execution time comparison of tools using benchmarks datasets

JOA ST and ISTF are run for both (1 node, 1 processor per node) and (1 node, 8 processors per node) settings. Average execution times are listed in Table 1. Running times are comparable and usage of more processors per node decreased the running time of JOA because of parallel implementation of ST and ISTF algorithms. For “5 small files”, BEDTools “multiinter” parameter usage increased its running time as it has a different implementation which is also reflected in its output. Number of overlapping intervals found for each benchmark dataset is listed in Table 5. JOA and BEDTools found exactly the same number of overlapping intervals whereas GROK and BEDOPS found different number of overlapping intervals. This might be because of a different design, implementation or a case which is handled differently such as nested intervals.

**Table 5:** Number of overlapping intervals found for each benchmark

Benchmarks	Number of overlapping intervals found				
	GROK	BEDOPS	BEDTools	JOA ST	JOA ISTF
2 small files	145925	145925	145925	145925	145925
5 small files	93080	93080	93080	93080	93080
(2.3M, 2.3M)	2972223	753639	13903684	13903684	13903684
(6.4M, 6.4M)	7369608	1292747	47936947	47936947	47936947
(9.2M, 9.2M)	11553395	3046101	39217773	39217773	39217773
(6.4M, 9.2M)	4746709	424074	19645676	19645676	19645676

JOA and BEDTools found exactly the same number of overlapping intervals whereas GROK and BEDOPS found different number of overlapping intervals

#### Execution time comparison of tools using semi-synthetic datasets

To compare the tools on a controlled dataset, we uniformly sampled 500bp long intervals from the human genome and generated bed format input. In the first scenario, we kept the number of intervals constant at 100,000 in each file and we increased the number of files by twofold from 2 to 512. In the second scenario, we kept the number of files constant at 2 and we increased the number of intervals in each file by twofold from 1,000,000 to 16,000,000.

In the firs scenario, we could not run GROK for more than 256 input files since calling a python function with more than 256 arguments was not allowed. Also, in the first scenario, BEDTools is called with the “multiinter” parameter when there are more than two input files.

Average execution times for both scenarios are provided in Tables 2 and 3. In the first scenario, when the number of input files is greater than 2, BEDTools average execution times are extremely high because of the “multiinter” option usage. In both scenarios, JOA has reduced execution times when 2 processors per node (ppn) is used instead of 1 ppn.

The number of overlapping intervals found for the first scenario and the second scenario are provided in Tables 6 and 7, respectively. JOA and BEDTools found the same number of overlapping intervals for the semi-synthetic datasets as in the case of benchmark datasets. However, GROK and BEDOPS found different number of overlapping intervals.

**Table 6:** Number of overlapping intervals found by the tools for the first scenario of semi-synthetic datasets

Simulations 1^*st*^ Scenario (#ofFiles,#ofIntervals)	Number of Overlapping Intervals Found				
	GROK	BEDOPS	BEDTools	JOA ST	JOA ISTF
(2,100000)	3958	3761	3913	3913	3913
(4,100000)	5	5	5	5	5
(8,100000)	0	0	0	0	0
(16,100000)	0	0	0	0	0
(32,100000)	0	0	0	0	0
(64,100000)	0	0	0	0	0
(128,100000)	0	0	0	0	0
(256,100000)	-	0	0	0	0
(512,100000)	-	0	0	0	0

**Table 7:** Number of overlapping intervals found by the tools for the second scenario of semi-synthetic datasets

Simulations 2^*nd*^ Scenario (#ofFiles,#ofIntervals)	Number of Overlapping Intervals Found				
	GROK	BEDOPS	BEDTools	JOA ST	JOA ISTF
(2,1M)	432665	280649	402931	402931	402931
(2,2M)	1741459	804859	1613107	1613107	1613107
(2,4M)	6509486	1785275	6455690	6455690	6455690
(2,8M)	20936796	2706065	25812190	25812190	25812190
(2,16M)	55354698	2336735	103245490	103245490	103245490

#### Execution time comparison of JOA ST versus ISTF using ENCODE data

To show the parallel processing advantage of JOA, we designed and ran an additional case study for JOA using 141 interval sets of Dnase hypersensitive sites. We found and supplied all jointly overlapping intervals for these 141 interval sets in Additional file 1: Table S1. This additional case study verified that JOA can handle a significantly larger number of interval files and JOA is able to attain better running times with the help of its parallel processing ability as shown in Table 4.

### JOA segment tree versus indexed segment tree forest detailed execution time comparison

**Fig. 2:**
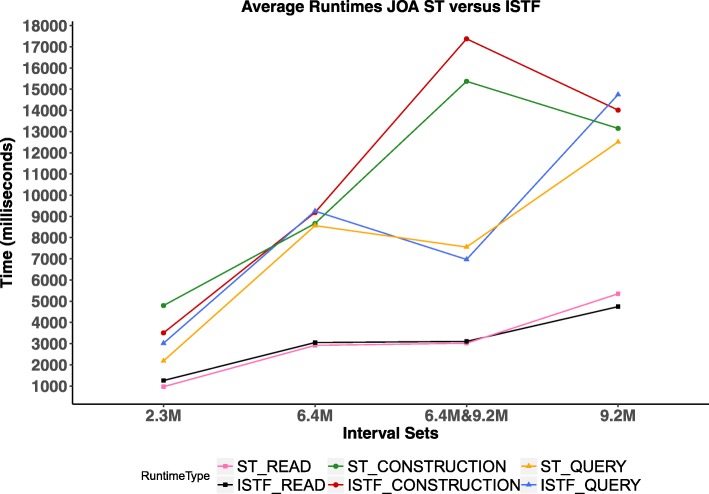
We compare JOA segment tree and indexed segment tree forest w.r.t. read, construction and query time which are averaged over 50 runs. This comparison shows that segment tree and indexed segment tree forest are comparable to each other

We used the last four benchmarks (2.3M, 2.3M), (6.4M, 6.4M), (9.2M, 9.2M), and (6.4M, 9.2M) from benchmarks datasets in Table 1. We compared JOA ST versus JOA ISTF with respect to read, construction and query running times which are averaged over 50 runs. We show that ST and ISTF perform comparably with respect to read, construction and query running times as it is shown in Fig. 2.

### JOA memory usage

Segment tree requires $\mathcal {O}(nlog{n})$ storage for *n* given intervals. Since indexed segment tree forest is based on indexing of segment tree nodes at a certain depth, it has similar memory requirements. JOA is implemented in Java with fork/join framework which speeds up parallel processing by using all available processor cores. However, parallel processing requires loading all the data into memory. Also, segment tree and indexed segment tree forest construction requires memory allocation of many temporary data structures which can be only deallocated automatically by the Java garbage collector.

We calculated the memory usage of JOA ST and ISTF for the semi-synthetic datasets. The memory usage in megabytes (MBs) are presented in Additional file 2: Tables S2 and S3. All the memory usage in the first and second scenarios are as expected; however, for the 2 interval sets of 8M intervals each, and for the 2 interval sets of 16M intervals in the second scenario, JOA’s memory consumption is high. This can be the due to the loading all interval sets into memory, the additional temporarily used data structures, or the Java garbage collector. As long as Java Virtual Machine has enough memory, garbage collector may not take action to deallocate memory and follow manually written memory deallocation statements in the code.

## Discussion

Parallel implementation of JOA ST and JOA ISTF algorithms require all the input intervals to be loaded into the memory. Moreover, JAVA garbage collector deallocates memory automatically without taking manually written memory deallocations into account. Therefore, although JOA ST and JOA ISTF have comparable and sometimes lower execution times, their memory footprint may be high.

**Fig. 3:**
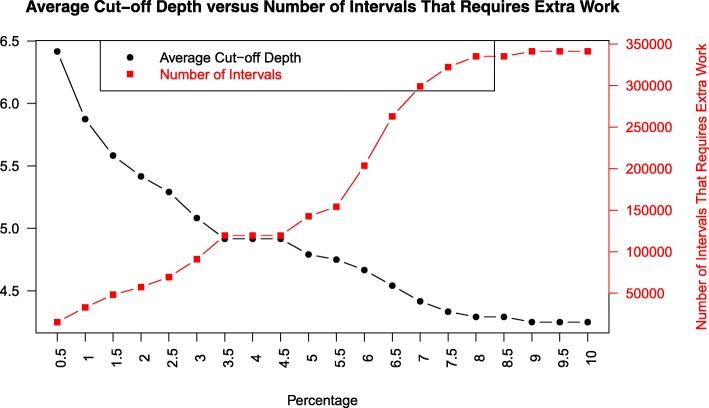
We analyze the effect of percentage parameter on the average cut-off depth and the number of intervals that require extra work. This analysis shows that as we increase the percentage parameter, as expected, the number of intervals that need to be moved increases and average cut-off depth decreases

**Fig. 4:**
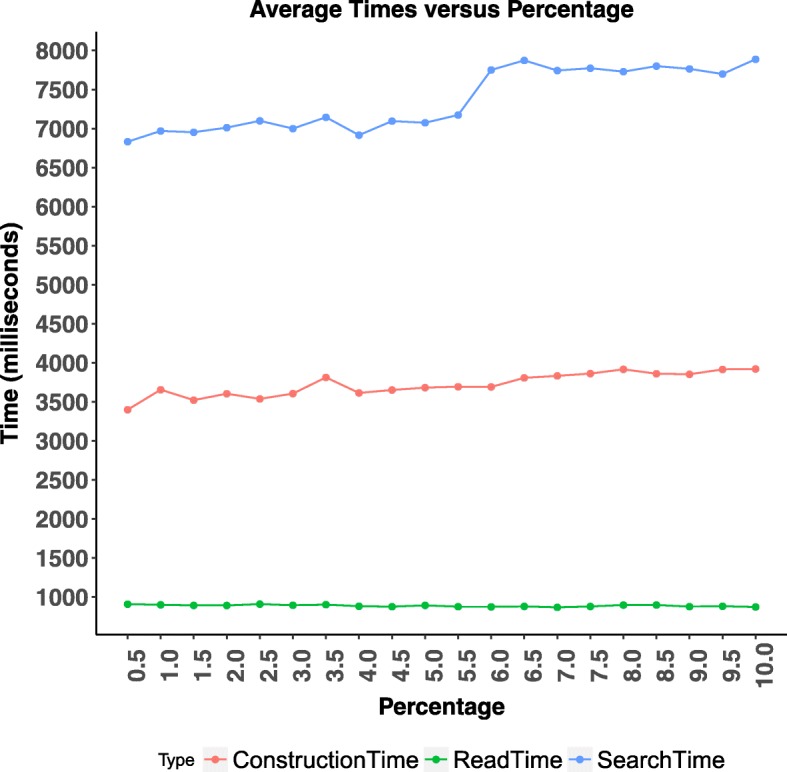
We analyze the effect of percentage parameter on the average runtimes. These runtimes are averaged over 50 runs using 2.3 million intervals files. This analysis shows that as we increase the percentage parameter, as expected, average read time does not change but the construction time and query time are increased

Regarding JOA ISTF, we analyzed how the percentage parameter affects the cut-off depth and how the cut-off depth affects the execution time (Figs. 3 and 4). Depending on these analyses, we set the percentage parameter to 0.5*%*. In addition to that, we provided a detailed analysis on the average height of the binary search trees (BSTs) constructed from hash indexes. We showed that average height of BSTs is always less than or equal the height of the original segment tree.

**Fig. 5:**
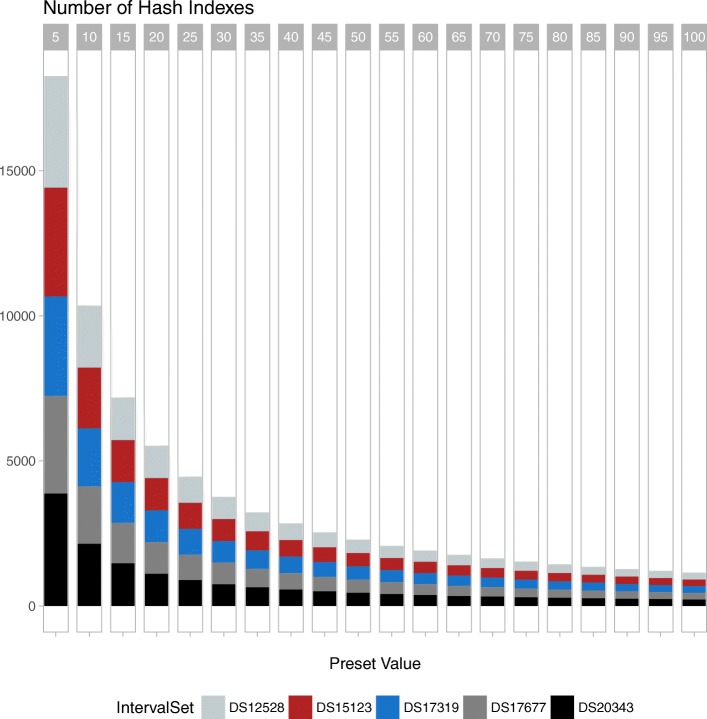
Number of hash indexes for varying preset values. Preset values must be multiplied by 10K

Furthermore, for JOA ISTF, we analyzed the number of hash indexes for varying preset values using human genome, chromosome 1 intervals of the 5 small files’ which is provided as the second benchmark dataset in Table 1. The smaller the preset value, the higher the number of different hash indexes, and vice versa (Fig. 5).

**Fig. 6:**
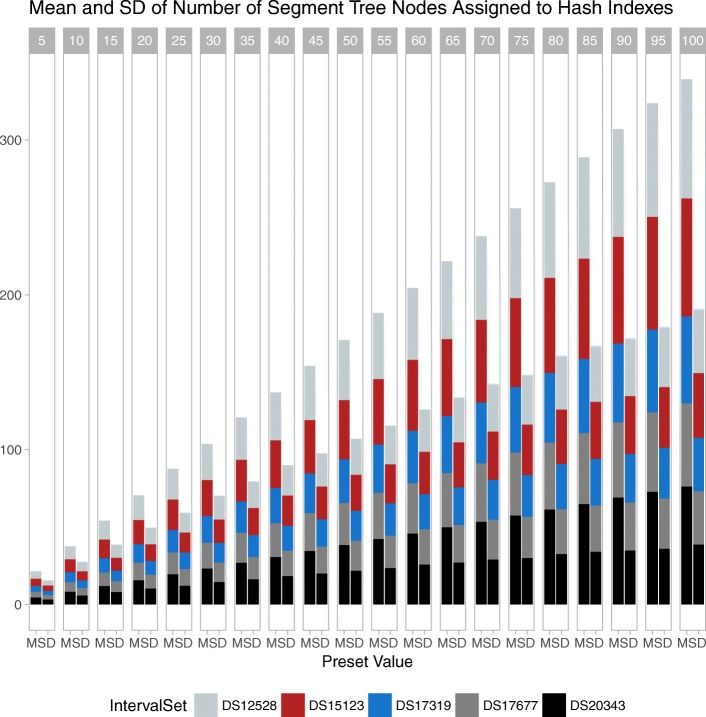
Mean and standard deviation of number of segment tree nodes assigned to hash indexes for varying preset values. Preset values must be multiplied by 10K

Moreover, we computed the mean and standard deviation of the number of segment tree nodes assigned to the same hash index as we vary the preset value. The smaller the preset value, the less the number of segment tree nodes assigned to a hash index (Fig. 6). This shows that there is a trade off between number of hash indexes and the mean number of segment tree nodes assigned to a hash index as we change the preset value. This is an inherent advantage of the proposed indexed segment tree forest construction algorithm and allows for generalizing to varying preset values.

## Conclusion

In this paper, we presented efficient methods for parallel joint overlap analysis of *n* interval sets. The proposed segment tree and novel indexed segment tree forest solutions are optimized with a divide and conquer algorithm design and implemented as a tool named JOA. We showed that JOA ST and ISTF have comparable execution times. We compared JOA with other state of the art tools such as GROK, BEDTools, BEDOPS and demonstrated the efficacy of JOA with respect to the execution time. Especially, when the number of processors per node is increased, JOA had less execution time than the other tools. Furthermore, we also verified that JOA is able to identify all the overlapping intervals.

To verify the parallel processing advantage of JOA, we designed and ran an additional case study for JOA in which we found the jointly overlapping intervals of 141 interval sets of ENCODE Dnase hypersensitive sites.

## Methods

### Segment tree

Similar to interval trees, segment tree data structure is another well-known space partitioning tree and a data structure for storing intervals. Its space complexity is $\mathcal {O}(nlog{n})$ and it can be constructed in $\mathcal {O}(nlog{n})$ time for *n* given intervals. Finding all intervals in the segment tree containing a query point *q*_*x*_ requires $\mathcal {O}(log{n}+k)$ time for *n* given intervals and *k* hits [14].

Let $I := {[x_{1} : x^{\prime }_{1}], [x_{2} : x^{\prime }_{2} ],..., [x_{n} : x^{\prime }_{n}]}$ be a set of *n* given intervals on the real line. Let *p*_1_,*p*_2_,...,*p*_*m*_ be the list of distinct interval endpoints (low and high endpoints), sorted from left to right. We simply partition the real line induced by these endpoints *p*_*i*_. We name the regions in this partitioning as elementary intervals. Thus, the elementary intervals from these endpoints *p*_1_, *p*_2_, …, *p*_*m*−1_, *p*_*m*_ are, from left to right, [*p*_1_:*p*_1_],(*p*_1_:*p*_2_),[*p*_2_:*p*_2_],...,(*p*_*m*−1_:*p*_*m*_),[*p*_*m*_:*p*_*m*_].

To this end, we build a binary search tree *T* whose leaves correspond to these elementary intervals induced by the endpoints of the intervals in *I* in an ordered way: the leftmost leaf corresponds to the leftmost elementary interval, and so on. We denote the elementary interval corresponding to a leaf *μ* by *Int*(*μ*).

An internal node of *T* represents the intervals that are the union of intervals of its two children: *Int*(*ν*) corresponding to node *ν* is the union of the intervals *Int*(*ν*_*leftChild*_) and *Int*(*ν*_*rightChild*_) in the subtree rooted at *ν*. The interval represented by the parent of leaf nodes, *Int*(*ν*), is the union of the elementary intervals *Int*(*ν*_*leftChild*_) and *Int*(*ν*_*rightChild*_). Note that elementary intervals are different from the input intervals, *I*.

**Fig. 7:**
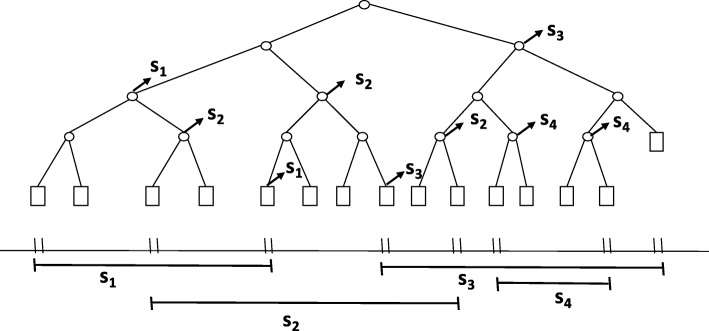
Intervals (*s*_1_,*s*_2_,*s*_3_,*s*_4_) are stored in the nodes. The arrows from the nodes point to their canonical subsets

Each internal node *ν* in *T* represents an interval *Int*(*ν*) whereas each leaf node *μ* represents an elementary interval, *Int*(*μ*). Each node stores a subset of input intervals, i.e., the canonical subset, *I*(*ν*), where *I*(*ν*)⊆*I*. This canonical subset of node *ν* stores the intervals [*x*:*x*^′^]∈*I* such that *Int*(*ν*)⊆[*x*:*x*^′^] and *Int*(*parent*(*ν*))⫅̸[*x*:*x*^′^]. This implies that if interval [*x*:*x*^′^] is in the canonical subset of node *ν*, *I*(*ν*), then *Int*(*ν*) is completely contained in [*x*:*x*^′^] whereas *Int*(*parent*(*ν*)) is not contained in [*x*:*x*^′^]. The constructed balanced binary tree *T* is called a segment tree. This way of construction ensures non-overlapping, totally consecutive intervals for the nodes at any depth, from left to right. In other words, it provides a natural binning at any depth of the tree. In Fig. 7, we exemplify how we store 4 intervals in the segment tree leaves and internal nodes which are constructed from the endpoints of these 4 given intervals. Moreover, in Fig. 7, the arrows from the nodes point to their canonical subsets.

#### Segment tree construction complexity analysis

To construct a segment tree for *n* given intervals, we proceed as follows: We sort the endpoints of *n* intervals in *O*(*n* log*n*) time and define elementary intervals at each end point and between each consecutive endpoints. We then construct a binary tree on these elementary intervals, where each interval is the union of its left and right child’s intervals (or elementary intervals). This process continues until the extent of all of the intervals are represented by the root node. The construction can be done bottom-up in linear time. In the last phase, *n* given intervals are stored in the canonical subset of the nodes, when a node’s interval, *Int*(*ν*), is fully contained in an input interval. As a result, an interval can be attached to more than one node and the number of intervals attached to nodes decreases as we go up in the tree; since, a node’s interval, *Int*(*ν*), becomes larger as we get closer to the root node.

### Segment tree query

A query is processed starting at the root node. If the query point *q*_*x*_ overlaps with the node’s interval, *Int*(*ν*), the associated intervals stored at that node, *I*(*ν*), are output and the query continues on the left or right child of that node, visiting one node per level of the tree. The time complexity of a segment tree query is *O*(log*n*+*k*) where *n* is the number of intervals and *k* is the number of overlapping intervals in the segment tree for the query point *q*_*x*_ [14].

### Indexed segment tree forest

After analyzing constructed segment trees for real data sets, we observed that nodes at the top of the segment tree (approximately top two thirds of the segment tree) do not store any intervals or store only a few intervals in their canonical subsets. In other words, input intervals are mostly stored in the bottom nodes of the segment tree.

Keeping the whole segment tree with a significant number of nodes without any or with a few intervals might be unnecessary. Furthermore, passing through all these nodes for each query in order to find overlapping intervals may increase the query time. Instead of having one tall segment tree, we can cut the segment tree at a certain depth close to the bottom of the tree and have as many short segment trees rooted at segment tree nodes present at this cut-off depth plus the segment tree nodes with no children above this cut-off depth.

### Indexed segment tree forest parameters

#### Cut-off depth

We decide on the cut-off depth by considering the total number of intervals stored in canonical subsets of nodes at the cut-off depth and above the cut-off depth. We find a depth, *d*, such that the number of intervals stored from root node to the nodes at this depth is greater than 0.5*%* of the total number of intervals stored in the segment tree. We then choose *d*+1 as the cut-off depth. The closer the cut-off depth to the bottom of the tree, the more segment trees there will be in the forest. In other words, cut-off depth determines the number of short segment trees in the forest.

**Moving intervals that were stored in the nodes above the cut-off depth:** All the intervals attached to the nodes that are above the cut-off depth must be distributed to the nodes at the cut-off depth. Specifically, if an interval is attached to a node above the cut-off depth, then this interval must be attached to its offspring nodes at the cut-off depth. If there is no offspring node at the cut-off depth then there can be two cases. Case 1: If the node storing the interval has no offspring, then we directly add this node to our segment tree forest. Case 2: If the node has offspring/s above the cut-off depth, then we attach the interval to its lowest offspring nodes and add these lowest offspring nodes to our segment tree forest, with a higher priority given to the node closer to the cut-off depth in order to keep the order consistent between the intervals of the nodes. Note that we do this extra work for a small number of nodes.

**Fig. 8:**
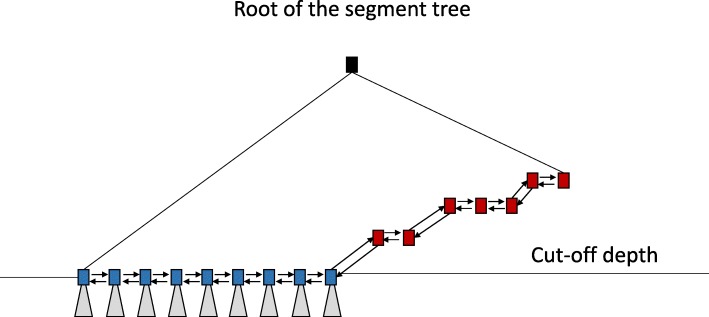
Segment tree nodes (blue) at the cut-off depth and segment tree nodes (red) with no children above the cut-off depth are stored in our segment tree forest. To enhance fast access, these stored segment tree nodes are connected to each other through forward and backward links

**Linking segment tree nodes at the cut-off depth to each other:** To ensure fast access between consecutive segment tree nodes at the cut-off depth, we connect segment tree nodes to each other through forward and backward pointers. We call these nodes linked nodes (See Fig. 8).

As mentioned earlier, we cut the segment tree at the cut-off depth and keep the segment tree nodes at this cut-off depth and the nodes with no children above the cut-off depth in an indexed segment tree forest. Each segment tree node at this cut-off depth is in fact a root of a segment tree in the forest and we compute its hash index using a hash function for each segment tree node and we store [index,segment tree node] tuples in a map.

#### Cut-off depth decision, percentage parameter

We make use of percentage parameter in cut-off depth decision. This percentage parameter determines the number of intervals that needs to be moved w.r.t. the total number of intervals stored in the segment tree. As this percentage parameter increases, the number of intervals that we accept to move increases, and we cut the original segment tree closer to the leaves. We analyzed how this percentage parameter affects the cut-off depth and how the cut-off depth affects the construction and query time. For this purpose, we used an input of 2.3 million intervals and varied the percentage parameter from 0.5 to 10.0. We observed that as we increase the percentage parameter, the number of intervals that need extra movement increases and the cut-off depth decreases, as expected (See Fig. 3). In this context, cut-off depth is defined as depth from the leaf level. However, construction and query time increases slightly, most probably because of more individual nodes are added to the indexed segment tree forest data structure (See Fig. 4). Based on these results, we decide on cut-off depth by considering movement of 0.5*%* of the total intervals. As a result, we internally set the percentage parameter to 0.5*%*.

#### Hash function, preset value

To index the nodes at the cut-off depth we use one universal hash function as shown in Eq. 1. By using this hash function, we index these short segment trees and we access each short segment tree in $\mathcal {O}(1)$ time instead of $\mathcal {O}(cut-off)$ time. The preset value in the hash function determines the number of different hash indexes that one can have.

1 $$  hashIndex = (node.interval.low/presetValue)  $$

**Fig. 9:**
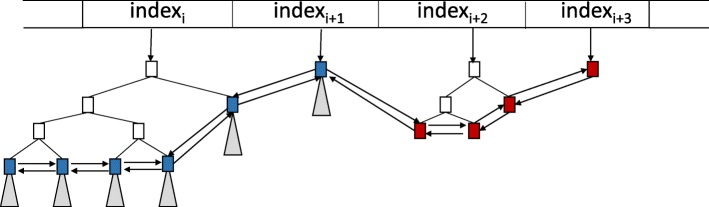
Segment tree nodes with the same index are stored in a BST and the root of BST is indexed. Blue and red colored nodes are original segment tree nodes which are linked to each other. Blue colored nodes are in fact the roots of the segment trees below them. Red colored nodes do not have any children. Parents of these blue and red colored nodes are the artificial nodes, if any

Lower preset values result in many hash indexes with fewer segment trees with the same index, therefore, fewer collisions. But, lower preset values may result in sequential search of more than one segment tree, which is definitely not preferred. Conversely, higher preset values result in fewer hash indexes with more segment tree nodes with the same index, which implies more collisions. To efficiently handle collisions, we construct a binary search tree (BST) from the segment tree nodes with the same index and store the root of this BST in the hash map. Parent nodes of these linked nodes in the BST constitute the artificial nodes as shown in Fig. 9.

This collision handling strategy implies that each segment tree may be reached in $\mathcal {O}(height(BST))$ time instead of $\mathcal {O}(height(OriginalSegmentTree))$ time. As long as the height of BST formed from these segment trees with the same index is less than the height of the original segment tree, search in indexed segment tree forest will be still less than search in one tall segment tree.

Theoretically, when all the nodes at the cut-off depth have the same hash index; the same segment tree will be constructed for them. Therefore, the height of the hash BST will be always less than or equal to the height of the original segment tree. To exemplify this situation, we carried out an analysis by using inputs of 2.3 million intervals. We observed that average height of the hash BST is less than the average height of the original segment tree for each chromosome. We also averaged over all chromosomes and showed that the average height of hash BSTs is 12.9 and the average height of the original segment tree is 19.4 for varying percentages from 0.5 to 10 and for the preset value of 1000000 as shown in Table 8.

**Table 8:** Average height of hash BSTs and original segment tree for all chromosomes are presented

	Average Height	
Percentage	Hash BSTs	Original Segment Tree
0.5	12.9695	19.4
1.0	12.9823	
1.5	12.9842	
2.0	12.9838	
2.5		
3.0	12.9842	
3.5	12.9845	
4.0		
4.5		
5.0	12.9843	
5.5		
6.0	12.9845	
6.5	12.9831	
7.0	12.9821	
7.5		
8.0	12.9814	
8.5		
9.0		
9.5		
10.0		

While preset value of 1000000 is kept constant, percentage parameter used in cut-off depth decision is varied from 0.5 to 10.0, and average height of hash BSTs and original segment tree are found using 2.3 million intervals files. It is observed that average height of hash BSTs are always less than the average height of the original segment tree

### Query in indexed segment tree forest

For each query interval, we compute its *lowIndex* and *highIndex* using its low and high endpoints, respectively. We start searching on a linked node pointed by the *lowIndex* if it exists, otherwise we find the *lowerIndex* (highest index lower than *lowIndex*) and start searching at the node shown by the *lowerIndex* and continue searching forward. If it is not possible, we start searching on the linked node pointed by the *highIndex* if it exists, if not, we compute *higherIndex* (lowest index higher than *highIndex*) and search the node pointed by *higherIndex* and continue searching backward. If there is no node pointed by *higherIndex*, it means that there is no overlapping interval with the query interval. The pseudocode of the indexed segment tree forest search algorithms are provided in the Additional file 2.

#### How to guarantee that at most two additional index searches are enough?

**Fig. 10:**
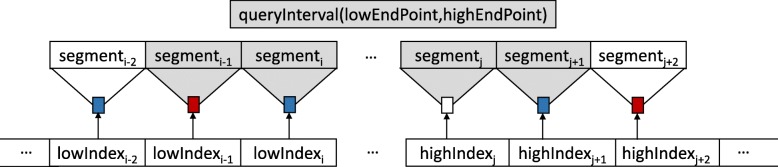
Searching the nodes pointed by *lowIndex*_*i*_ and *highIndex*_*j*_, the nodes in between them, and plus two more nodes at most is enough

As it is shown in Fig. 10, we first compute *lowIndex*_*i*_ and *highIndex*_*j*_ using query low and high endpoints, respectively. Then we search for the segment trees pointed by one of these indexes in the order of *lowIndex*_*i*_, *lowIndex*_*i*−1_, *highIndex*_*j*_ or *highIndex*_*j*+1_.

Here we present why we may need to consider only two more segment trees pointed by the indexes *lowIndex*_*i*−1_ and the *highIndex*_*j*+1_ (Fig. 10).

2 $$ {lowIndex}_{i}=queryLow/presetValue  $$

3 $$ {highIndex}_{j}=queryHigh/presetValue  $$

4 $$ {lowIndex}_{i-2} < {lowIndex}_{i-1} < {lowIndex}_{i}  $$

5 $$ {lowIndex}_{i-1} < {lowIndex}_{i} \Rightarrow  $$

6 $$  {lowNode}_{i-1}.interval.low < queryLow  $$

From the preserved order between intervals of consecutive nodes we know that

7 $$ \begin{aligned} {lowNode}_{i-2}.interval.high < \\ {lowNode}_{i-1}.interval.low \end{aligned}  $$

Equations 6 and 7 imply that

8 $$ {lowNode}_{i-2}.interval.high < queryLow   $$

As a result of inequality Eq. 8, *lowNode*_*i*−2_.*interval* and query interval can not overlap. Therefore we may need to look at only one more index preceding the *lowIndex*_*i*_ and search for the segment tree pointed by that index and forward. In the same manner, we may need to consider only one more index subsequent to the *highIndex*_*j*_ and search for the segment tree pointed by that index and backward.

### Real datasets: benchmarks

We defined six benchmarks, which are listed in Table 1 for execution time comparisons of the tools.

The first two benchmarks include five files containing DNase-Hypersensitivity hotspot peaks from mouse fetal tissues: fAdrenal-DS12528, fAdrenal-DS15123, fAdrenal-DS17319, fAdrenal-DS17677 and fAdrenal-DS20343. They contain 193835, 188966, 137386, 132500, and 195098 intervals, respectively. The first benchmark that is called “2 small files” is a subset containing the first two of the five files, whereas the second benchmark named as “5 small files” is the set of all five files.

The last four benchmarks come from three files consisting of histone modifications from human cell lines: HepG2, Dnd41 and A459 including 2,362,386 (2.3M), 6,473,749 (6.4M), and 9,218,913 (9.2M) intervals, respectively. The last benchmark is a subset containing the last two of these files. Sources of these benchmark datasets are provided in Availability of data and material.

## Additional files

Additional file 1 Supplementary Table S1 contains all jointly overlapping intervals for 141 interval sets of Dnase hypersensitive sites. (XLSX 75 kb)

Additional file 2 Supplementary Material includes Supplementary Tables S2 and S3 for memory usage of JOA ST and ISTF for the semi-synthetic datasets. Supplementary Material also provides the pseudocode of the indexed segment tree forest search algorithms. (PDF 135 kb)

## Abbreviations

NGS: Next Generation Sequencing
JOA: Joint Overlap Analysis
ST: Segment Tree
ISTF: Indexed Segment Tree Forest
