# Supplementary material for: JOA: Joint Overlap Analysis of multiple genomic interval sets
Source: BMC Bioinformatics. 2019 Mar 8;20:121. doi: 10.1186/s12859-019-2698-4 (PMC6408804; doi:10.1186/s12859-019-2698-4)
Supplement: Supplementary file 2 — Supplementary Material includes Supplementary Tables S2 and S3 for memory usage of JOA ST and ISTF for the semi-synthetic datasets. Supplementary Material also provides the pseudocode of the indexed segment tree forest search algorithms. (PDF 135 kb) [file 12859_2019_2698_MOESM2_ESM.pdf]

Supplementary Materials for 'JOA: Joint Overlap Analysis of Multiple Genomic Interval Sets'

Burçak Otlu[1], & Tolga Can[1]
[1] Department of Computer Engineering, Middle East Technical University, Ankara, Turkey

January 25, 2019

1 Indexed Segment Tree Forest Search Algorithms

Algorithm 1: search

```
Require: queryIntervals
Require: index2NodeMap
Require: overlappingIntervalsList
qOvIntList : queryOverlappingIntervalsList
for each query interval do
   qOvIntList ← mainSearch(query, index2NodeMap, presetValue)
   update overlappingIntervalsList with qOvIntList
end for
```

Algorithm 2: mainSearch

```
Require: query(lowEndPoint, highEndPoint)
Require: index2NodeMap
Require: presetValue > 0
overlappingIntervals ← ∅
lowIndex ← lowEndPoint/presetValue
highIndex ← highEndPoint/presetValue
lowNode ← index2NodeMap.get(lowIndex)
if lowNode ≠ null and linked(lowNode) then
   searchAtLinkedNode(lowNode, query, overlappingIntervals)
else if lowNode ≠ null then
   if overlaps(query, lowNode) then
      searchDownward(lowNode, query, overlappingIntervals)
   end if
   rightNode ← findRightMostLnkd(lowNode)
   searchForward(rightNode.forwardNode, query, overlappingIntervals)
   leftNode ← findLeftMostLnkd(lowNode)
   searchBackward(leftNode.backwardNode, query, overlappingIntervals)
else
   lowerIndex ← getLowerIndex(index2NodeMap, lowIndex)
   lowerNode = index2NodeMap.get(lowerIndex)
   if lowerNode ≠ null then
      searchAtLowerNode(lowerNode, query, overlappingIntervals)
   else
      highNode ← index2NodeMap.get(highIndex)
      if highNode ≠ null and linked(highNode) then
         searchAtLinkedNode(highNode, query, overlappingIntervals)
      else if highNode ≠ null then
         if overlaps(query, highNode) then
            searchDownward(highNode, query, overlappingIntervals)
         end if
         rightNode ← findRightMostLnkd(highNode)
         searchForward(rightNode.forwardNode, query, overlappingIntervals)
         leftNode ← findLeftMostLnkd(highNode)
         searchBackward(leftNode.backwardNode, query, overlappingIntervals)
      else
         higherIndex ← getHigherIndex(index2NodeMap, highIndex)
         higherNode = index2NodeMap.get(higherIndex)
         if higherNode ≠ null then
            searchAtHigherNode(higherNode, query, overlappingIntervals)
         end if
      end if
   end if
end if
return overlappingIntervals
```

Algorithm 3: searchAtLinkedNode

```
Require: node is a linked original node
Require: query(lowEndPoint, highEndPoint)
Require: overlappingIntervals
searchForward(node, query, overlappingIntervals)
searchBackward(node.backwardNode, query, overlappingIntervals)
```

Algorithm 4: searchForward

```
Require: node is a linked original node
Require: query(lowEndPoint, highEndPoint)
Require: overlappingIntervals
low: lowEndPoint
high: highEndPoint
if node ≠ null and node.interval.low ≤ high then
   if low ≤ node.interval.high then
      add node.canonicalSubset to overlappingIntervals
      if node.left ≠ null and low ≤ node.left.interval.high then
         searchDownward(node.left, query, overlappingIntervals)
      end if
      if node.right ≠ null and node.right.interval.low ≤ high then
         searchDownward(node.right, query, overlappingIntervals)
      end if
   end if
   searchForward(node.forwardNode, query, overlappingIntervals)
end if
```

Algorithm 5: searchBackward

```
Require: node is a linked original node
Require: query(lowEndPoint, highEndPoint)
Require: overlappingIntervals
low: lowEndPoint
high: highEndPoint
if node ≠ null and low ≤ node.interval.high then
   if node.interval.low ≤ high then
      add node.canonicalSubset to overlappingIntervals
      if node.left ≠ null and low ≤ node.left.interval.high then
         searchDownward(node.left, query, overlappingIntervals)
      end if
      if node.right ≠ null and node.right.interval.low ≤ high then
         searchDownward(node.right, query, overlappingIntervals)
      end if
   end if
   searchBackward(node.backwardNode, query, overlappingIntervals)
end if
```

Algorithm 6: searchDownward

```
Require: query(lowEndPoint, highEndPoint)
Require: node ≠ null
Require: node and query overlaps
Require: overlappingIntervals
low: lowEndPoint
high: highEndPoint
Add node.canonicalSubset to overlappingIntervals
if node.left ≠ null and low ≤ node.left.interval.high then
   searchDownward(node.left, query, overlappingIntervals)
end if
if node.right ≠ null and node.right.interval.low ≤ high then
   searchDownward(node.right, query, overlappingIntervals)
end if
```

Algorithm 7: searchAtLowerNode

```
Require: lowerNode ≠ null
Require: query(lowEndPoint, highEndPoint)
Require: overlappingIntervals
if linked(lowerNode) then
    searchForward(lowerNode, query, overlappingIntervals)
else
    if overlaps(query, lowerNode) then
        searchDownward(lowerNode, query, overlappingIntervals)
    end if
    node ← findRightMostNode(lowerNode)
    searchForward(node.forwardNode, query, overlappingIntervals)
end if
```

Algorithm 8: searchAtHigherNode

```
Require: higherNode ≠ null
Require: query(lowEndPoint, highEndPoint)
Require: overlappingIntervals
if linked(higherNode) then
    searchBackward(higherNode, query, overlappingIntervals)
else
    if overlaps(query, higherNode) then
        searchDownward(higherNode, query, overlappingIntervals)
    end if
    node ← findLeftMostNode(higherNode)
    searchBackward(node.backwardNode, query, overlappingIntervals)
end if
```

2 Jointly Overlapping Intervals for 141 ENCODE Dnase Hypersensitive Sites

Supplementary Table 1: An additional case study for JOA using 141 ENCODE Dnase hypersensitive sites. We found and supplied all jointly overlapping intervals for these 141 Dnase hypesensitive sites files in. Excel file: SupplementaryTable1.xlsx

3 JOA Memory Usage

Simulations 1st Scenario (#ofFiles,#ofIntervals)	Memory Usage in MBs	
	JOA ST	JOA ISTF
(2,100000)	149	160
(4,100000)	297	321
(8,100000)	97	117
(16,100000)	84	115
(32,100000)	80	236
(64,100000)	551	401
(128,100000)	518	268
(256,100000)	380	135
(512,100000)	604	594

Supplementary Table 2. Memory Usage of JOA in MBs for the 1st scenario simulated datasets.

Simulations 2nd Scenario (#ofFiles,#ofIntervals)	Memory Usage in MBs	
	JOA ST	JOA ISTF
(2,1M)	935	975
(2,2M)	1894	1944
(2,4M)	4171	4210
(2,8M)	9039	9179
(2,16M)	21343	18659

Supplementary Table 3. Memory Usage of JOA in MBs for the 2nd scenario simulated datasets.
